# Measurement and Exposure Assessment of Intermediate Frequency Magnetic Fields From Electronic Article Surveillance (EAS) Gates in Libraries

**DOI:** 10.3389/fpubh.2022.871134

**Published:** 2022-05-12

**Authors:** Miwa Ikuyo, Kaoru Esaki, Atsuko Aimoto, Kanako Wake, Sachiko Yamaguchi-Sekino, Noriko Kojimahara, Yukihisa Suzuki, Masao Taki

**Affiliations:** ^1^Department of Systems Design, Tokyo Metropolitan University, Hachioji, Japan; ^2^Electromagnetic Compatibility Laboratory, National Institute of Information and Communications Technology, Koganei, Japan; ^3^Work Environment Research Group, National Institute of Occupational Safety and Health, Kawasaki, Japan; ^4^Epidemiology, Shizuoka Graduate University of Public Health, Shizuoka, Japan

**Keywords:** electromagnetic field (EMF), exposure assessment, electronic article surveillance (EAS) gate, dosimetry, induced electric field, ellipticity, library worker, epidemiology

## Abstract

Exposure to magnetic fields from the electronic article surveillance (EAS) gate was evaluated in consideration of the application to epidemiological studies of library workers who are exposed continually to intermediate frequency magnetic fields from the EAS gate. Two types of exposures were investigated. One was transient exposure due to passing through or beside the gate and another was chronic exposure in the room. We measured magnetic fields from five EAS gate models which were commonly used in libraries in Japan. Detailed measurements were performed for two of them in consideration of the phase difference of vector components of magnetic flux density. The polarization of the magnetic field in the gate was investigated with the index of ellipticity. The induced electric field in a human body was numerically calculated for exposures to magnetic fields of the two gate models. The results provide a quantitative understanding of exposures during passing through or by the EAS gate. Magnetic field distribution was measured in a large room for one gate model to quantify the chronic exposure of library workers during the work at the desk. It was found that the magnetic field was distributed as a function of the horizontal distance to the nearest gatepost. The 45-point average value *B*_IEC_ defined by the IEC standard was suggested to be a useful quantity to characterize the magnitude of the magnetic field from the EAS gate. Exposures to different EAS gates are expected to be compared through this quantity without detailed measurements. These results are expected to provide useful means for exposure assessment of epidemiological studies on the association between the IF-EMF exposure and possible health outcomes.

## Introduction

It has long been pointed out that studies have not been done sufficiently on the health effects of exposure to electromagnetic fields (EMF) in the frequency range from 300 Hz to 10 MHz (referred to as the Intermediate Frequency (IF) range) compared to extremely low frequency (ELF) and radiofrequency (RF) ranges (1, 2), especially epidemiological studies on IF-EMF are needed, but are not yet sufficient (3, 4). On the other hand, the opportunities for exposure to IF-EMF in daily lives are expected to increase with the spread of wireless power transfer (WPT) for electric vehicles soon. Therefore, epidemiological studies for risk assessment of IF-EMF are urgently needed. This paper deals with the exposure assessment for IF-EMF as a key issue for the epidemiological study.

The types of IF-EMF equipment and their magnetic field levels have been reported by a systematic review paper (5). The occupational exposures to IF-EMF were investigated by the development of a source-based measurement database (6) and source-exposure matrix (7). Also, the measured IF-EMF levels emitted from a wide variety of home appliances have been reported (8, 9). These studies show that one of the devices that causes high human exposure to IF-EMF is the walk-through gate for the Electronic Article Surveillance (EAS) systems. As mentioned in the report of the International Commission on Non-Ionizing Radiation Protection (ICNIRP) (10), several types of EAS technology have achieved significant market success and represent the vast majority of installed systems. Among them, our research is focused on the EAS gate using “electromagnetic” technology (referred to as EM-EAS). This category of EAS systems has been used commonly in libraries in Japan.

Exposure to magnetic fields emitted from EAS gates is divided into two types: one is the exposure of people going through the gate or passing beside the gatepost, and the other is the exposure of library workers who work long hours in the surrounding space of the gate. The former, as stated in the ICNIRP Statement (11), is the main factor of human exposure to IF-EMF generated by security devices, and is being focused on in previous studies. The latter is a factor related to occupational exposure, and the ICNIRP Statement (11) pointed out that exposure time can be extended to full working hours at the longest. However, no paper has ever examined this kind of long-time exposure to relatively low-level IF-EMF among library workers sitting at some distance from the gate.

The magnetic fields emitted from the EM-EAS gates are spatially non-uniform, sinusoidal continuous waves of several hundred Hz to several tens of kHz. Assessment of magnetic fields from EAS gates was carried out in Sweden (12) showing that the arithmetic means of measured magnetic fields according to CENELEC standard (13) exceeded the ICNIRP 1998 reference level (14) for all the EM-EAS gates investigated. Similar excesses were reported in subsequent investigations (15, 16). These measurements also showed that the magnetic fields were significantly higher in the proximity to the gatepost than in the middle of the gate aisle. Assessment of the induced electric field in the body is needed to ensure conformity with the basic restrictions in the proximity to the gatepost. Some dosimetric research has been performed on EM-EAS gates (17, 18), but they used the simulated magnetic fields generated by a model of a gatepost consisting of two coils. In addition, they did not take the elliptical polarization of the magnetic field due to the phase difference of coil currents into account.

In this study, we measured IF magnetic fields from five EM-EAS gate models which are commonly used in libraries in Japan. Detailed measurements were performed for two of them in consideration of the phase difference of vector components of magnetic flux density. Characteristics of polarization of magnetic field were investigated in terms of ellipticity. Comparison of exposures was performed between exposures during passing through the gate and passing beside the gatepost.

The results are compared with exposure guidelines. An exposure index named exposure ratio (ER) is quantified for the exposures from the EAS gates. We discuss the application of our results to exposure assessment for an epidemiological study on IF-EMF exposures of library workers.

## Materials and Methods

### Investigated EAS Gates

We first performed a preliminary survey of university libraries in Japan to identify commonly used EAS gates. The survey suggested that most of the university libraries with EAS gates are equipped with EM-EAS gates. We selected five models with relatively high adoption rates. These five models cover about 90% of the EAS gates used in university libraries in Japan.

Table 1 summarizes the frequency and external specifications of the investigated five EM-EAS gates G1–G5. It should be noted that the operating frequency ranges from 220 Hz to 14 kHz and the frequencies of G4 and G5 are 220 Hz, which is not in the IF region (> 300 Hz). Those are included in this study considering the common nature of the exposure.

**Table 1:** Specifications of investigated EM-EAS gates.

**EM-EAS Gate**	**Operating frequency**	**Sizes of gatepost H × W × D [cm^**3**^]**	**Distance between gateposts [cm]**
G1	366 Hz	180 × 79 × 10	90
G2	14 kHz	178 × 66 × 6.4	91
G3	366 Hz	149 × 73 × 2.0	90
G4	220 Hz	178 × 65 × 6.4	90
G5	220 Hz	180 × 66 × 8.8	100

Those five models have built-in coils (current-carrying coils) inside the gateposts. The coils are excited by continuous sinusoidal currents to generate sinusoidal magnetic fields of continuous waves. Although their frequencies are different as shown in Table 1, the magnetic fields generated by the coils have similar characteristics regarding spatial distributions. Normally, two gateposts are erected vertically, facing each other at a predetermined interval. The monitoring zone is formed between the gateposts to detect the magnetic tags.

### Measurement of Magnetic Fields

#### Measurement Equipment

We measured the magnetic fields from the EAS gates using a magnetic field meter (Model FT3470; Hioki, Japan) with an isotropic 3-axis probe (3 cm^2^ sensor). The measured waveforms were stored in a data acquisition oscilloscope (MEMORY HiCORDER MR8847A; Hioki, Japan).

Magnetic fields generated by the EM-EAS gates are sinusoidal IF-EMF. As several coils are driven by a current with different phases, the magnetic fields are not necessarily linearly polarized but they can be elliptically polarized. In this case, the phase of each vector component is different from each other. Therefore, it is necessary to measure the phases of each vector component. For this reason, a phase reference magnetic field was measured at a fixed point using another magnetic field sensor (Custom-made by Hotonikusu, Japan). The measured components of the magnetic field vector are represented by complex quantities (phasors) with absolute value for the amplitude in root-mean-square (rms) value and argument for the phase.

#### Measurement Zones

**Figure 1:**
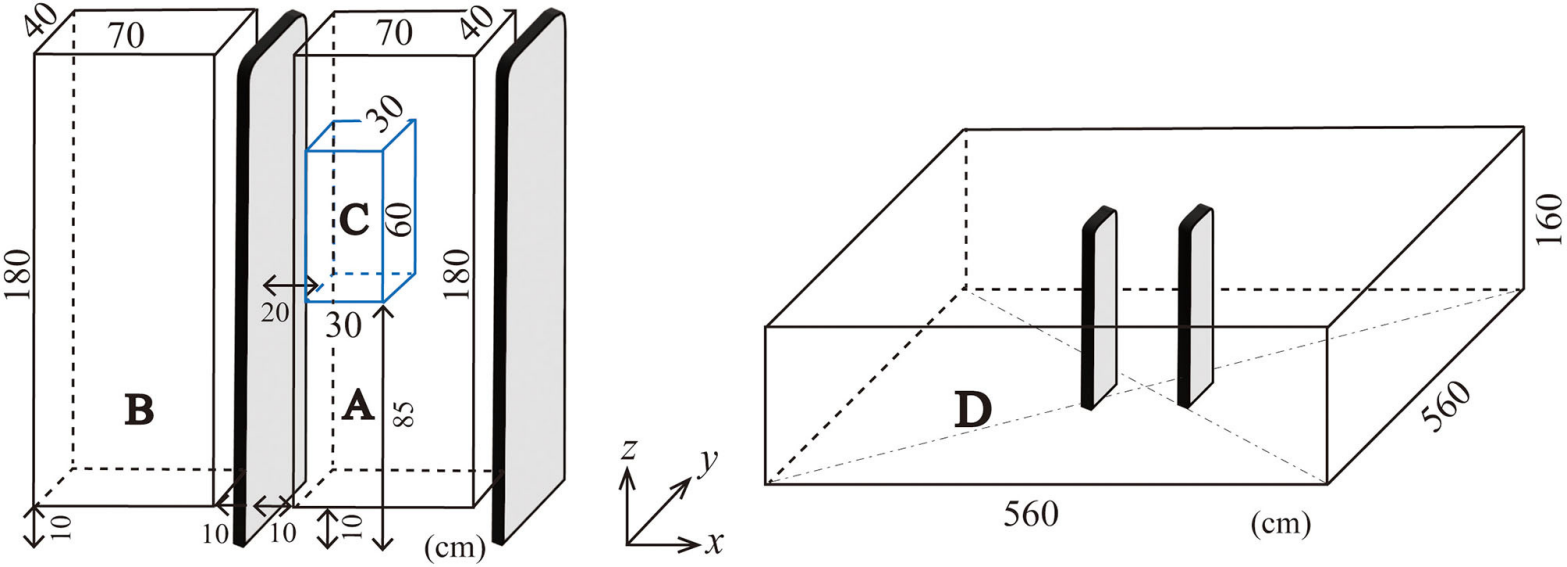
Illustration of measurement zones.

Measurements were performed in four zones A–D shown in Figure 1. Zone A is inside the EAS gate aisle. Exposure of a person passing through the gate is evaluated in zone A. Zone B is similar to zone A in size but just outside of the gatepost. Exposure of a person passing beside the gate is evaluated in zone B. Zone C is a limited region to evaluate human exposure in a way specified in an international standard of IEC (19) for evaluation of human exposure to electromagnetic fields from short-range devices including EAS gates. The same method is also standardized as a European Norm (20). Zone D is a larger space to evaluate the exposure of a person staying in a room where an EAS gate is equipped.

The locations of each zone, measurement intervals, and numbers of measurement points are summarized in Table 2. Zones A and B are defined to contain a whole human body to allow the analysis of induced electric field in a human body passing through (zone A) or by (zone B) the gate. Measurement grid intervals in zones A and B are 10 cm and the total number of measurement points are 760 for each zone.

**Table 2:** Measurement intervals and numbers measurement points in each zone.

**Location**	**Zone**	**Interval of measurement points [cm]**	**Number of measurement points**	**Measured gates**
Between gateposts	A	10	760	G1, G2
Beside of gatepost	B	10	760	G2
Specified by IEC 62369-1	C	15	45	G1– G5
Surrounding EAS gate	D	10–40	3564	G2

**Figure 2:**
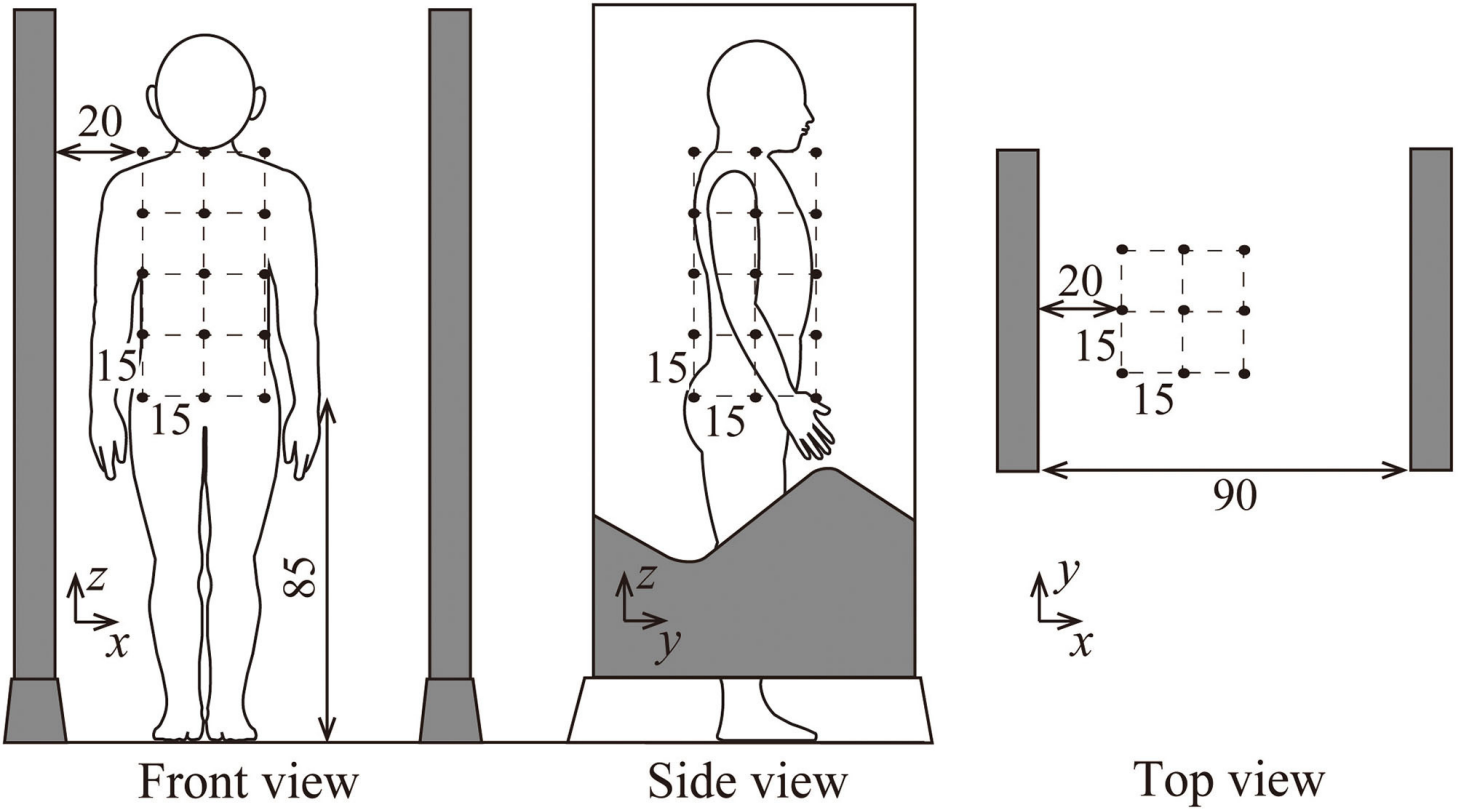
illustration of the grid points specified by IEC 62369-1 for an EAS gate consisting of dual floor standing antenna, a typical EAS gate in libraries.

Zone C is a sub-region of zone A for the spatial measurement according to the standards (19, 20). Those standards specify 45 (=3 x 3 x 5) measurement points with 15 cm grid intervals. The arithmetic mean of the 45 measurements is used for comparison with the ICNIRP reference level (14, 21), which is supposed to be compared with the spatial average of the magnetic field strength in a space to be occupied by an entire human body. The procedure is useful in the case when the magnetic field strength locally exceeds the reference level but the spatial average is expected below the reference level. Measurements at 45 points are practically feasible rather than averaging the incident magnetic field in the entire human body, or dosimetric evaluation based on the basic restrictions. Figure 2 illustrates the grid points specified for an EAS gate consisting of a dual floor standing antenna (19), a typical EAS gate in libraries.

Zone D covers a large space surrounding a pair of EAS gateposts. The grid intervals range from 10 to 40 cm. Grid intervals of 10 cm are applied near the gate. Grid intervals of 20 or 40 cm are applied in the rather distant regions. The total number of measurement points is 3,564.

Only one model G2 was allowed measurements in detail as the model was installed in our laboratory for test. The magnetic field of G2 was measured in all zones A–D. Another model G1 was also investigated in detail as it was installed in our university library. Measurements for G2 were carried out in zones A and C. Other models were not allowed to investigate in detail. Only IEC measurement (in zone C) and measurements at several selected points were carried out for the other three models (G3–G5).

#### Measurement Procedure

All measurements were performed manually using the 3-axis measurement system mentioned above and a wooden positioning device. Zones A and B are close to the multiple coils of the EAS gatepost which are excited by sinusoidal currents with different phases. Therefore, the phase of each vector component needs to be measured in the measurements in zones A and B.

The sinusoidal magnetic field vector rotates elliptically when the phases of the vector components are different. The elliptically polarized magnetic field is characterized by an index of ellipticity that is defined by the ratio of the short axis to the long axis of the orbit of the magnetic field vector. The ellipticity is obtained by Equation (1).


(1) $$\begin{array}{l} \frac{B_{min}}{B_{max}}=\sqrt{\frac{\left(B_{x}^{2}+B_{y}^{2}+B_{z}^{2}\right)-\sqrt{\alpha^{2}+\beta^{2}}}{\left(B_{x}^{2}+B_{y}^{2}+B_{z}^{2}\right)+\sqrt{\alpha^{2}+\beta^{2}}}} \end{array}$$

$$\begin{array}{l} \alpha =B_{x}^{2}\cos2\theta_{x}+B_{y}^{2}\cos2\theta_{y}+B_{z}^{2}\cos2\theta_{z}\\ \beta =B_{x}^{2}\sin2\theta_{x}+B_{y}^{2}\sin2\theta_{y}+B_{z}^{2}\sin2\theta_{z} \end{array}$$

where *B*_*min*_ and *B*_*max*_ are the rms values of the semi-minor and semi-major axes of the field ellipse, respectively. *B*_*x*_, *B*_*y*_, *B*_*z*_, θ_*x*_, θ_*y*_, θ_*z*_ are the rms amplitude and the phases of phasor vector components of the magnetic flux density, respectively.

The magnitude of the magnetic field is evaluated by the resultant rms value *B*_R_ (22) obtained by Equation (2).


(2) $$\begin{array}{l} B_{R}=\sqrt{B_{max}^{2}+B_{min}^{2}}=\sqrt{B_{x}^{2}+B_{y}^{2}+B_{z}^{2}} \end{array}$$

Measurement in zone C was performed according to the standardized procedure (19). The resultant rms values of magnetic flux density were measured at 45 grid points in zone C. Then the average of the resultant rms values measured at the 45 points was calculated to obtain an estimate of spatially averaged magnetic flux density in the space where a human body is assumed to occupy.

The estimate of the spatial average obtained from 45-point average is named IEC average *B*_IEC_ in this paper. The values of *B*_IEC_ were obtained for all EAS gates G1–G5.

Measurement in zone D covers a large space and the number of measurement points is very large. Only resultant rms values were of interest in this measurement as magnetic fields are almost linearly polarized in the region distant from the coils of the EAS gate. The measurement was performed only for G2. Limited numbers of measurements were performed in zone D for G3 (30 points) and G4 (10 points) to examine the consistency of the spatial distribution of the magnetic fields from different EAS gates.

### Calculation of the Induced Electric Field Strength Inside a Human Body

#### Numerical Method and Human Model

The magnetic fields emitted from EAS gates are spatially non-uniform and can locally exceed the reference levels of exposure guidelines in terms of magnetic flux density. The spatial average of incident magnetic field may be used as a next step to compare with the reference level, but it may still exceed the reference level in some cases. Induced electric field should be evaluated in those cases to compare with the basic restrictions.

The induced electric field in tissue was calculated using an anatomical voxel human model TARO developed by the National Institute of Information and Communications Technology (NICT) (23), The model consists of 51 tissues with a spatial resolution of 2 mm. The total number of voxels is about 8 million. The electric constants of those tissues were derived from the publicized database (24).

The EAS gates investigated emit magnetic fields of frequencies from 220 Hz to 14 kHz. A quasi-static approximation is applied in this case and the impedance method (25) was employed for the calculation of induced electric field in tissues. The impedance method is formulated based on Kirchhoff's loop law (or second law). Measured magnetic field data can be directly used to provide electromotive force in the loop with Faraday's law of induction. This is an advantage over the SPFD method which requires the calculation of vector potential from the measured data of magnetic fields (26).

It should be noted that the incident magnetic fields are elliptically polarized near the gatepost. The orientation of magnetic fields incident on the body varies with time in this case. The numerical calculations were performed using phasors as variables for magnetic flux density and induced electric field to deal with elliptically polarized fields.

#### Exposure Conditions

Induced electric fields are calculated for a human model passing through the gate assuming a human model standing in zone A. We also calculated a human model passing beside the gatepost assuming a human model standing in zone B. The human model was assumed to stand at a distance of 10 cm to the surface of the gatepost from the nearest part of the human model (arm) in zone A (inside of the aisle) or zone B (outside of the aisle).

The movement of the human body passing the gate causes additional time derivative of the magnetic field to induce additional electric field in tissue. This effect is ignored here as the time derivative due to the movement is negligibly small compared to the sinusoidal change with time at intermediate frequencies.

## Results

The data in the following sub-sections are summarized in Tables 3, 4. The list of symbols is given in Table 5.

**Table 3:** Summary of exposure characteristics.

**EAS Gate**	**G1**	**G2**	**G3**	**G4**	**G5**
*f* [Hz]	366	14 k	366	220	220
*B_*R*_* [μT] in zone A max./average/min.	430/100/30	439/128/19	–	–	–
*B_*inc*_ [* μT] in human body max./average/min.	181/68/30	282/88/19	–	–	–
*B*_IEC_ [μT] for zone C	87	111	68	87	105
*E_*in*_* [V/ m] in human body p99 (p99.9)	0.017 (0.032)	0.88 (1.72)	–	–	–
Exposure ratios to guidelines for general public exposure					
B_RL(g)_ [μT]	200	27	200	200	200
*ER*__*RL*_(g)_: *B*_IEC_ */* B_RL(g)_ [%]	44	411	34	44	53
E_BR(g)_ [V/ m]	0.4	1.9	0.4	0.4	0.4
*ER*__*BR*_(g)_: *E_*in*_* / *E*__*BR*_(g)_ [%] p99 (p99.9)	4.3 (8.0)	47 (91)	-	-	-
Exposure ratios to guidelines for occupational exposure					
B_RL(o)_[μT]	820	100	820	1000	1000
*ER*__*RL*_(o)_: *B*_IEC_ */* B_RL(o)_[%]	11	111	8.2	8.7	11
E_BR(o)_ [V/ m]	0.8	3.8	0.8	0.8	0.8
*ER*_BR(o)_: *E_*in*_*/ E_BR(o)_ [%] p99 (p99.9)	2.1 (4.0)	23 (46)	-	-	-

**Table 4:** Comparison of exposures in human bodies standing in the aisle (zone A) and beside the gatepost (zone B) of EAS gate G2.

**Measurement zone**		**zone A**	**zone B**
*B_*R*_* [μT] max./average/min (in zone)		439/128/19	371/70/8
*B_*inc*_* [μT] max./average/min (in human)		282/88/19	365/74/8
*E_*in*_* [V/ m]	p99 (p99.9)	0.88 (1.72)	0.95 (1.57)
*ER*_BR_ [%]	general public exposure (BR=1.89 V/ m) p99 (p99.9)	47 (91)	50 (100)
	occupational exposure (BR=3.78 V/ m) p99 (p99.9)	23 (46)	25 (50)

**Table 5:** List of quantities with descriptions.

**Quantity**	**Description**
**Measurement**	
*B_*R*_* [μT]	Resultant magnetic flux density in rms value.
*B_*inc*_* [μT]	Incident magnetic flux density in the space where human body is supposed to occupy.
*B*_IEC_ [μT]	Average of *B_*R*_* measured at the 45 points according to IEC62369-1.
**Dosimetry**	
*E_*in*_* [V/ m]	Dosimetric quantity of induced electric field inside the human body exposed to magnetic fields.
**ICNIRP guidelines 2010 and exposure ratio**	
B_RL(g)_ [μT]	Corresponding reference level in terms of magnetic flux density for general public exposure.
E_BR(g)_ [V/ m]	Corresponding basic restriction in terms of induced electric field for general public exposure.
B_RL(o)_[μT]	Corresponding reference level in terms of magnetic flux density for occupational exposure.
E_BR(o)_ [V/ m]	Corresponding basic restriction in terms of induced electric field for occupational exposure.

### Magnetic Field Near the Gate

**Figure 3:**
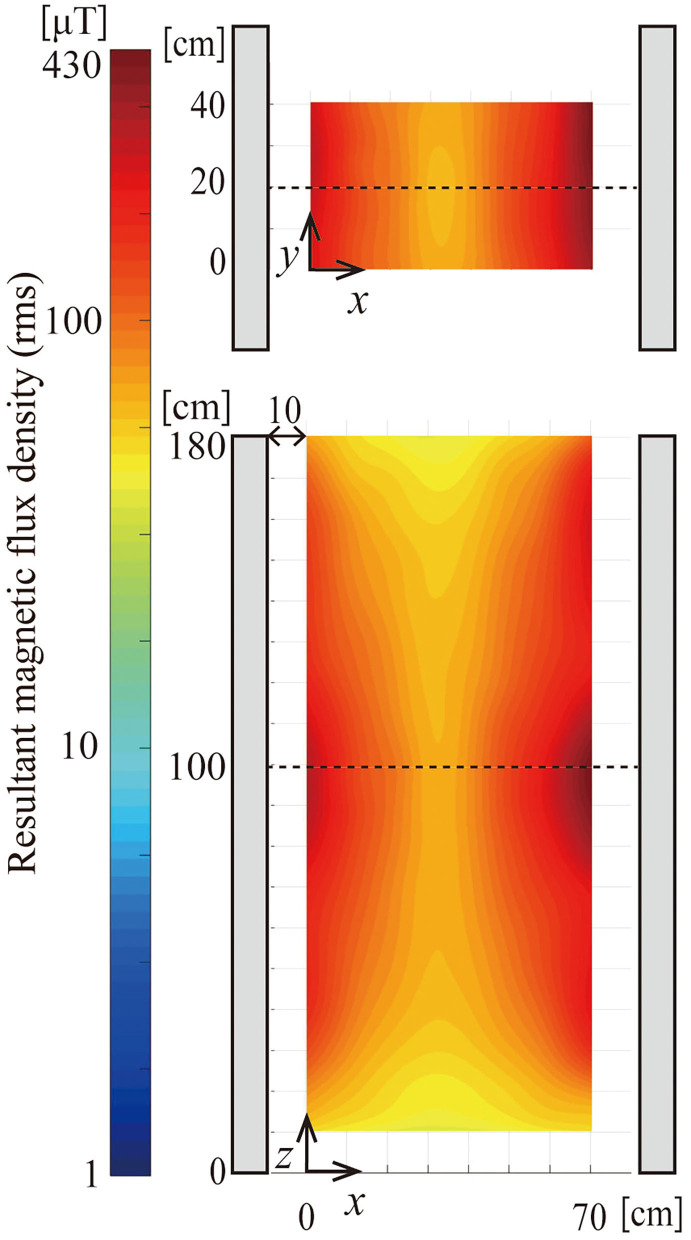
Distribution of magnetic field of EAS gate G1 (366 Hz) in zone A. Magnetic field was expressed in resultant rms value of magnetic flux density. The *xy*-cut is at *z* = 100 cm and the *xz*-cut is at *y* = 20 cm.

It was confirmed by the measurement that the waveforms of magnetic fields from EAS gates are continuous sinusoidal waves at frequencies nearly equal to the nominal frequencies shown in Table 1. Figure 3 shows the distribution of the resultant rms values of magnetic flux density for G1 (366 Hz) in zone A. Measured points were on the 10 cm grid and the measured data were interpolated to 2 mm resolution using bi-cubic interpolation to apply to dosimetry calculations with 2 mm resolution. The maximum was 430 μT, the average 100 μT, and the minimum 30 μT in zone A. The maximum, average, and minimum for G2 (14 kHz) in zone A were also obtained and were 439, 128, 19 μT, respectively.

**Figure 4:**
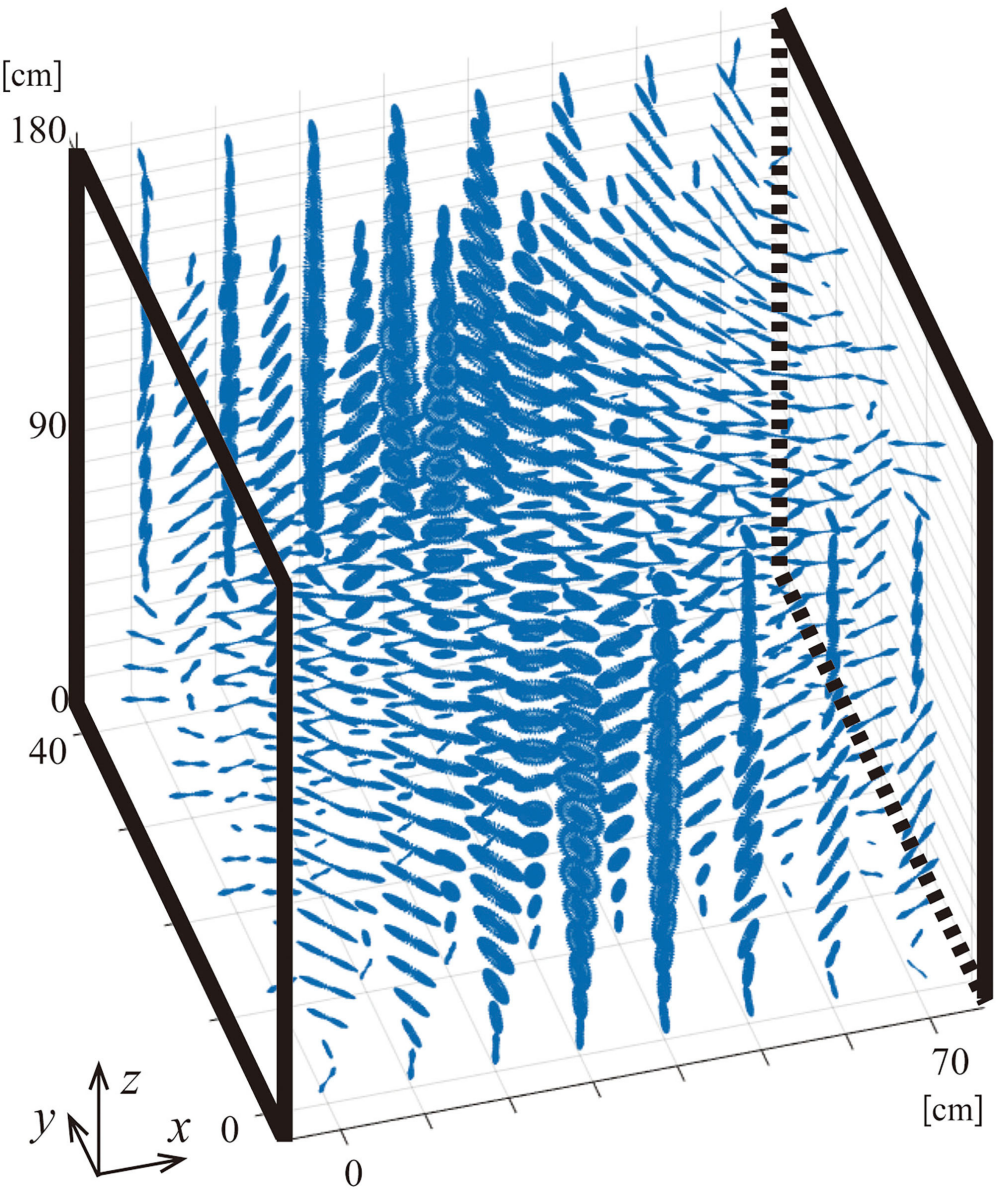
Illustration of elliptical rotation of magnetic field vectors in the gate aisle of G1. The scales in each direction of *x, y, z* are not the same to clarify the distribution of rotations.

Figure 4 illustrates ellipticity in zone A for G1. The ellipticity is larger near the center of the gate while it is small (or close to linearly polarized) near the gatepost (see also **Figure 8**). It should be noted that significant ellipticity is found only in the region where the magnetic flux density is rather small.

Measurements were carried out for G2 in zone B, or just outside of the gate aisle. The maximum, minimum, and average values were 371, 70, 8 μT, respectively. It was suggested that similar exposure occurs either passing through the aisle or passing by the gatepost.

### IEC Average

The IEC averages for G1–G5 were 87, 111, 68, 87, and 105 μT, respectively. The ratios to ICNIRP reference levels range from 34 to 411 % for the general public and 8.2–111 % for occupational exposures as shown in Table 3. The results confirmed that EM-EAS gates are sources of significantly strong IF-EMF encountered in daily lives.

### Magnetic Field Distribution in a Large Space

**Figure 5:**
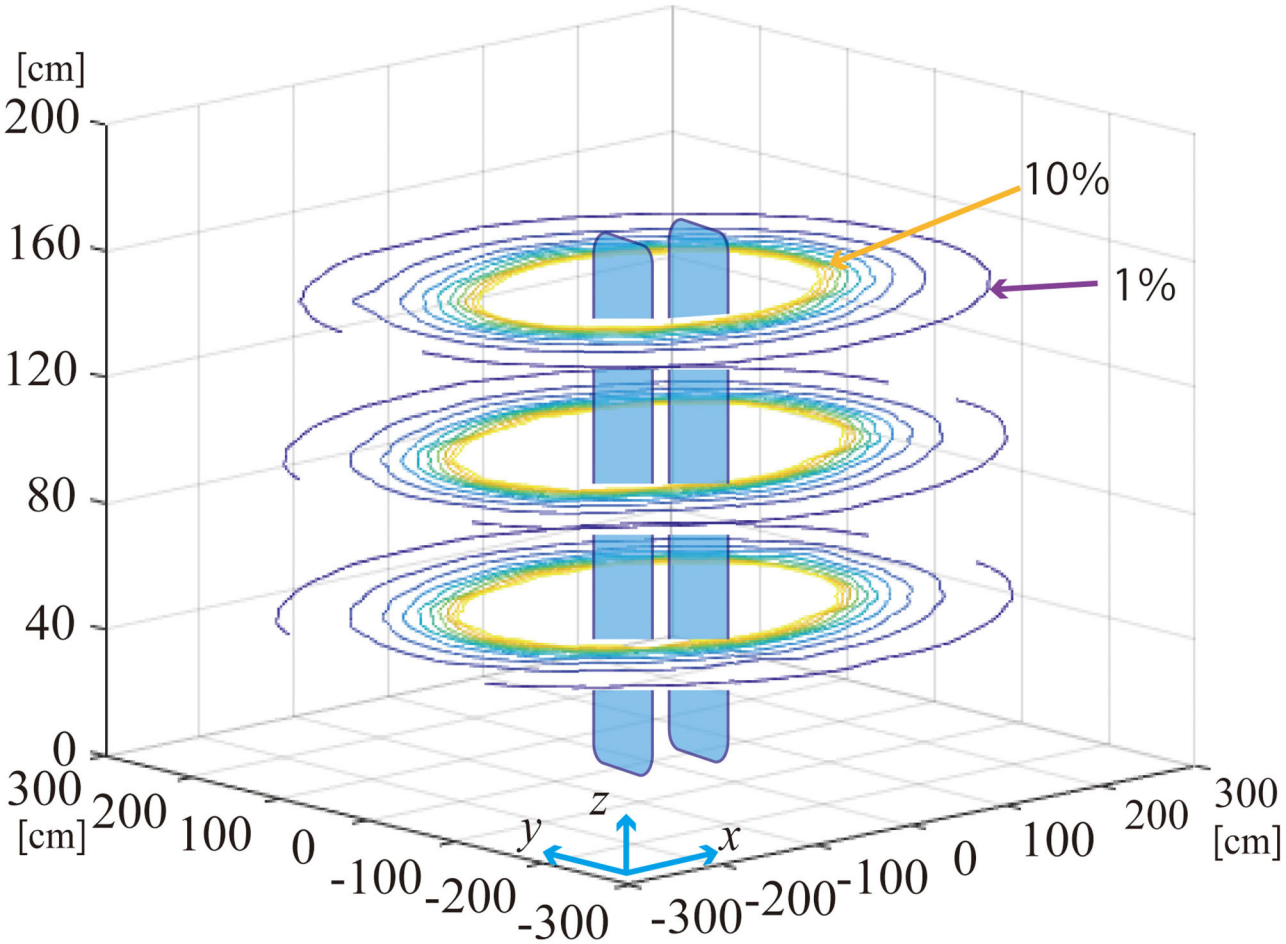
Contour map of magnetic flux density (resultant rms value) surrounding EAS gate G2 in zone D.

Figure 5 shows the distribution of rms magnetic flux density in zone D for G2 in contour lines. The magnetic flux density is approximately constant in the vertical direction and decays steeply with distance.

**Figure 6:**
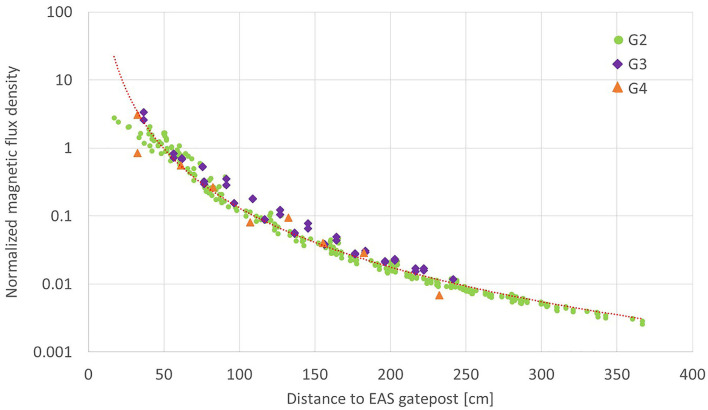
Dependency of magnetic flux density on the distance to the nearest gatepost. The values are measured for G2, G3, and G4, and they are normalized by *B*_IEC_ of each gate to allow comparison between different gates.

Dependency on the distance to the nearest gatepost is shown in Figure 6. The magnetic flux density is normalized by the IEC average *B*_IEC_ to allow comparison with different gates. The normalized magnetic flux density depends similarly on the distance to the nearest gatepost regardless of the direction. The dependency is approximated by Equation (3).


(3) $$\begin{array}{l} \frac{B_{R}\left(r\right)}{B_{IEC}}=7.8\times 10^{4}{\;r}^{-2.9} \end{array}$$

where *r* is the distance to the nearest gatepost in cm and *B*_*R*_(*r*) is the resultant magnetic flux density at the distance *r*. The coefficient of determination *R*^2^ is 0.976 for this approximation. The approximation is valid in the region *r* > 50 cm. The magnetic flux density at *r* = 50 cm is nearly equal to *B*_IEC_. It decays to 13% of *B*_IEC_ at 1 m from the nearest gatepost. It becomes 1.7 % at 2 m, and 0.6 % at 3 m distance from the nearest gatepost.

The data measured *in situ* for G3 and G4 are superimposed on Figure 6. It is found that the magnetic flux density normalized by *B*_IEC_ is well approximated by Equation (3) for different EAS gate models.

Equation 3 suggests that magnetic field from EAS gates decays in proportion to the approximately third power of the distance. The dependency is similar to the magnetic field from an equivalent dipole source (27). The approximation holds in the region *r* > 50 cm while the source is larger than the distance where the approximation holds. It is not natural for the large current source to be equivalent to a dipole source having the third power decay, but the relationship holds for the data measured for three different EAS gates consisting of two gateposts. The approximation formula is empirically obtained from a limited number of samples but it may be useful for estimation of exposure in a room equipped with EAS gates. It should be noted that the dependency is well approximated by Equation 3 when the distance is measured to the closer post of the pair of gateposts.

### Induced Electric Field

**Figure 7:**
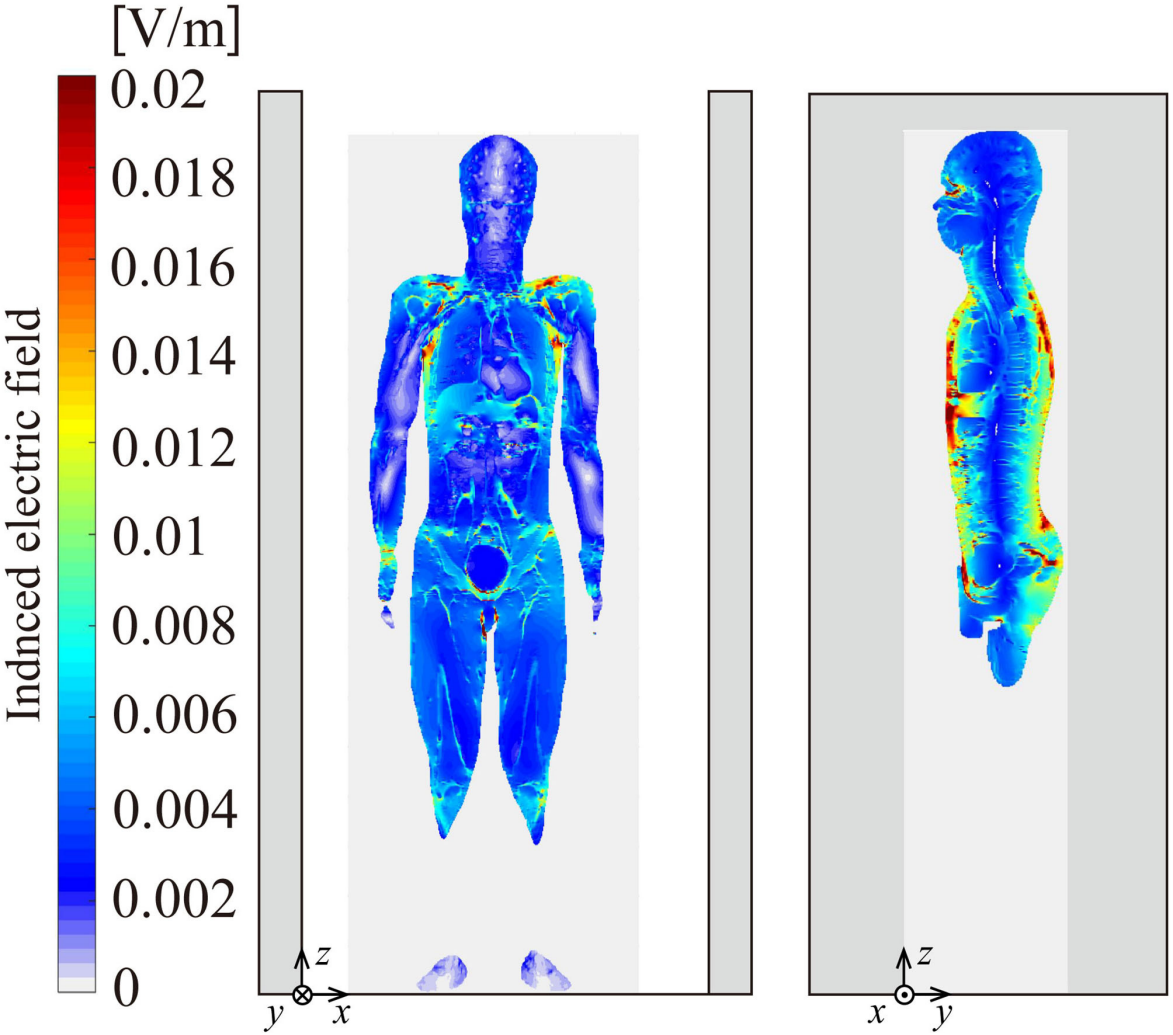
Distribution of induced electric field on the mid-coronal section (left) and the mid-sagittal section (right). The human model is standing in the gate aisle of G1.

Figure 7 shows the calculated result of induced electric field *E*_*in*_ in the human body passing through gate G1. The human model is located in zone A. The 99th and 99.9th percentile values (p99 and p99.9) in the whole body were 0.017 and 0.032 V/m, respectively, for G1. The p99 and p99.9 for G2 were 0.88 and 1.72 V/m, respectively. The values will be discussed in the next section.

**Figure 8:**
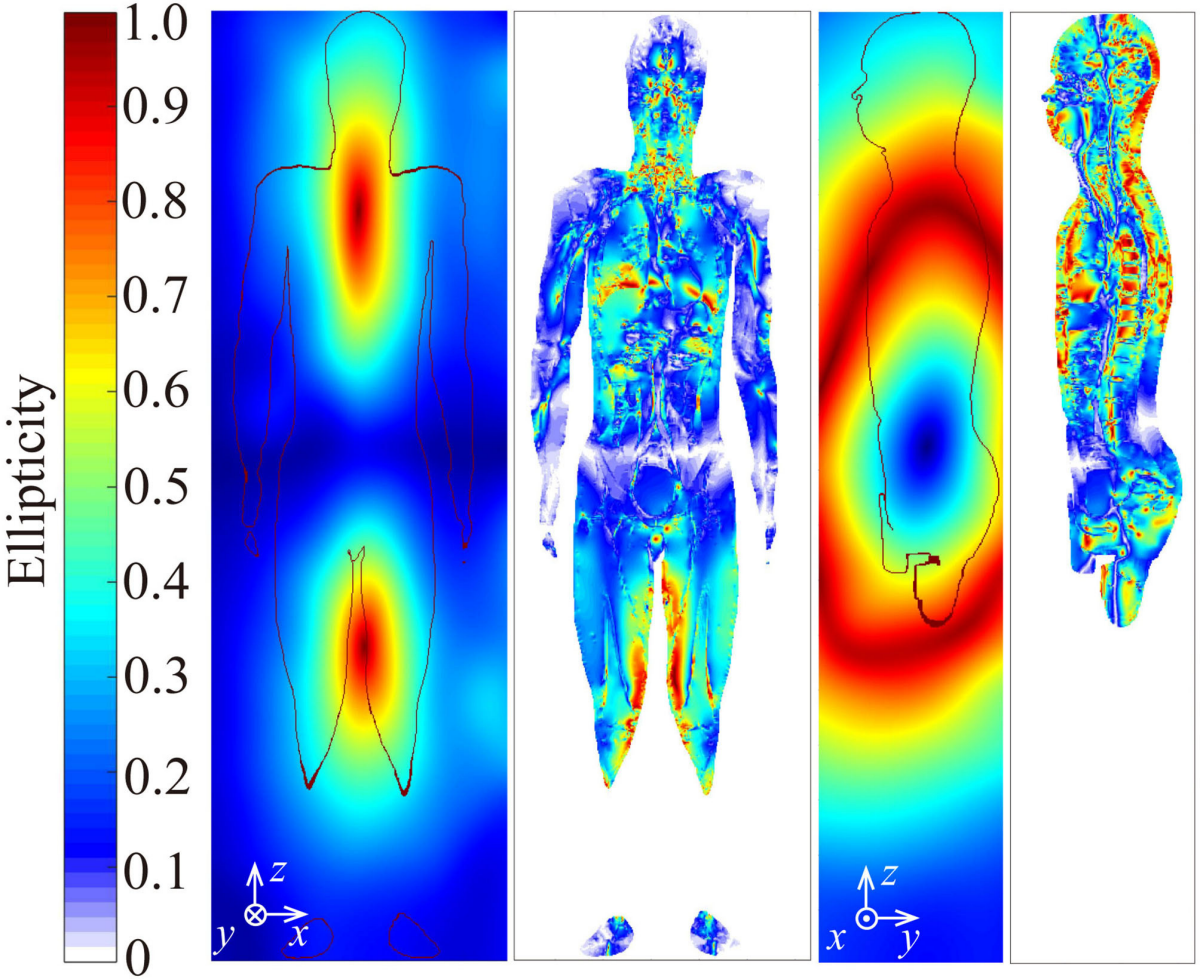
Ellipticity of incident magnetic field (left of each) and induced electric field (right of each) on the mid-coronal section and mid-sagittal section of the human model standing in the gate aisle of G1.

The magnetic fields from the EM-EAS gates are not linearly but elliptically polarized. Hence, the induced electric field in the body should also be elliptically polarized. Figure 8 shows the distributions of the ellipticity of incident magnetic flux density (left) and the ellipticity of induced electric field (right) in the mid-coronal and mid-sagittal sections of the body. It is confirmed that an elliptically polarized electric field is induced in tissue.

**Figure 9:**
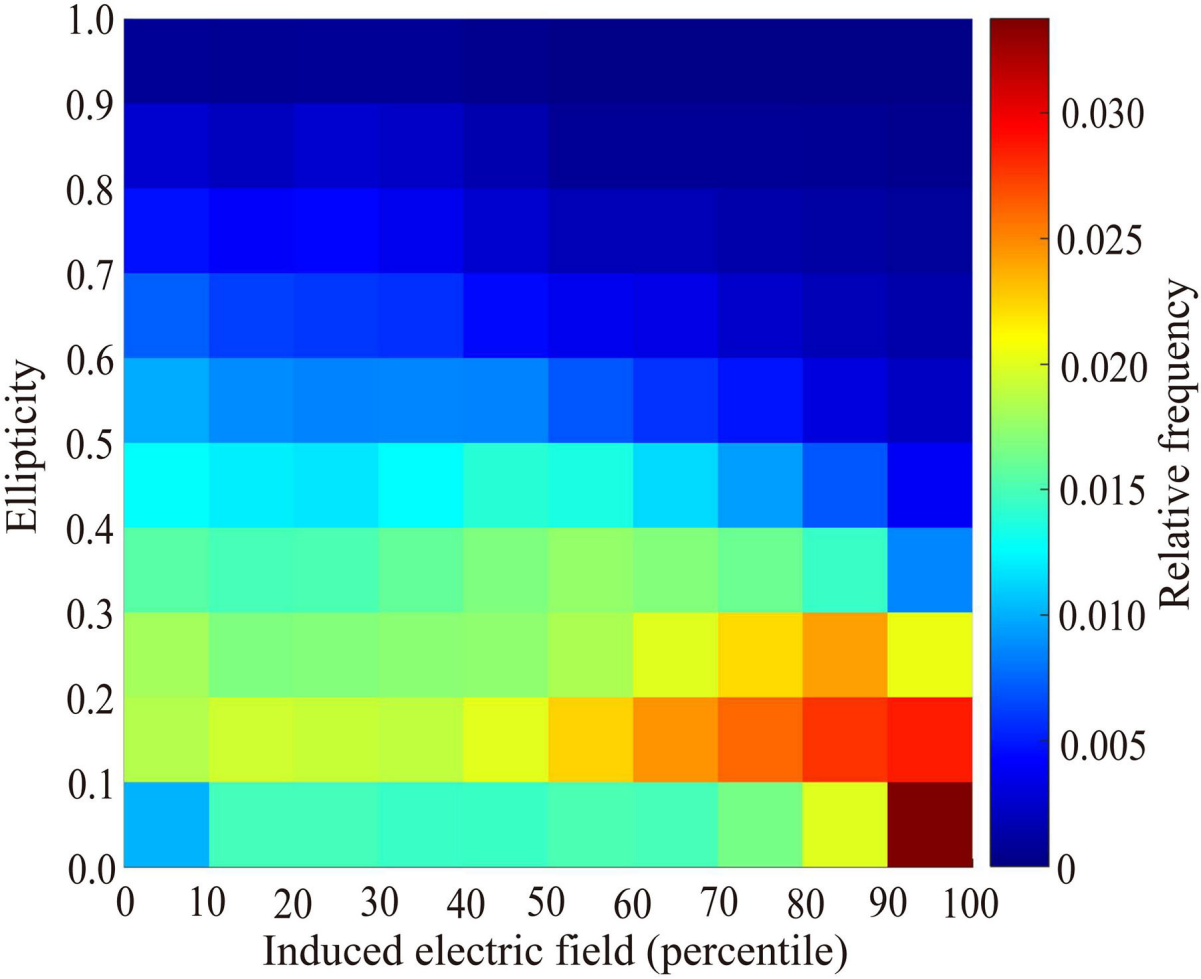
Distribution of relative frequency of voxels in the human model as a function of induced electric field and ellipticity in decile scales.

Figure 9 shows the frequency of ellipticity in voxels in the human model as a function of ellipticity and induced resultant electric field in the decile scale. Small ellipticity is significantly frequent in the 10th decile of the induced electric field in the tissue. This means that ellipticity is rather small in the tissue where the induced electric field is large.

The induced electric field is also calculated for a human passing beside the gate in zone B for G2 to compare the cases between passing inside and outside of the gate aisle. The p99 and p99.9 values were 0.95 and 1.57 V/m, respectively, for zone B while they were 0.88 and 1.72 V/m for zone A as mentioned above. The results suggested that exposure is almost of the same level in terms of the induced electric field between the cases during passing inside and outside of the gate aisle. The detailed comparison between exposures in zones A and zone B for G2 is shown in Table 4.

## Discussion

### Comparison of Induced Electric Field Between Different Gates

The induced electric field is proportional to both frequency and magnitude of the magnetic field (represented by *B*_IEC_). Then, the induced electric field is represented by Equation (4).


(4) $$\begin{array}{l} E_{in\;}=kfB_{IEC} \end{array}$$

where *k* is a constant, *f* is the operating frequency in Hz, and *B*_IEC_ is the IEC average in μT of the gate of interest. The proportional constant *k* corresponding to, for example, the p99 is expected to be close to each other assuming that the spatial distributions of incident magnetic flux density are similar for different gates and the electric constants of tissues are not significantly dependent on frequency. We have calculated the p99 of induced electric fields *E*_in_ = 0.017 V/m for G1 (*f* = 366 Hz, *B*_IEC_=87 μT) and *E*_in_= 0.88 V/m for G2 (*f* = 14 kHz, *B*_IEC_=111 μT). Then the constant *k* for the p99 is obtained as 5.34 × 10^−7^ (V m^−1^Hz^−1^μT^−1^) from the values for G1, and 5.66 × 10^−7^ from the values for G2. There is a 6% difference between the estimated *k* values but they are reasonably close. The result suggests that the maximum (e.g., p99) induced electric field should be roughly estimated for different EAS gates without detailed measurements and dosimetry calculations if *B*_IEC_ is obtained. It should be noted that the constant *k* may not be so close if the configuration of the coils is different from each other.

### Comparison With Exposure Guidelines

The exposure levels near the gates are compared with the ICNIRP guidelines (21). Table 3 summarizes the obtained data and results of the comparison. Table 3 also includes the spatial maximum/average/minimum of incident magnetic flux density *B*_*inc*_, that is, magnetic flux density in the space where the human body is supposed to occupy. The human body is assumed here to be at the location described in **2.3.2** in zone A. The average of *B*_*inc*_ is the quantity to be compared with the reference level for non-uniform EMF exposures.

Regarding the reference levels, the maximum magnetic flux density in zone A exceeds the reference level for the general public both for G1 and G2. The spatial average of incident field *B*_*inc*_ for G1 does not exceed the reference level but that of G2 still exceeds the reference level for the general public.

*B*_IEC_ is a quantity to be used for compliance assessment instead of the spatial average of *B*_*inc*_ according to the standardized assessment procedure. The measured values of *B*_IEC_ for G1 and G2 are both close to but sufficiently larger (i.e., conservative) than the average of *B*_*inc*_. This suggests that *B*_IEC_ is a useful quantity to evaluate exposure due to EAS gates. For G3– G5, the values of *B*_IEC_ are well below the reference level.

We defined exposure ratio to reference level (*ER*_*RL*_) as *B*_IEC_ */* B_RL_,where B_RL_ is the reference level of ICNIRP guidelines (21). This index is useful for whole-body uniform exposure but not relevant for localized exposures such as exposure of humans passing the EAS gate. The exposure ratio of G2 to the reference level exceeded 100% of the reference levels for both general public and occupational exposures.

We defined the exposure ratio to basic restriction (*ER*_*BR*_) as *E*_*in*_ / E_BR_. This index is useful to evaluate exposures including localized exposures. The exposure ratios of G1 and G2 to basic restriction did not exceed 100% of the basic restrictions for both general public and occupational exposures.

It was shown that magnetic fields from EAS gates are elliptically rotating fields. It should be noted that the orientation of the magnetic field relative to the human body changes with time. This characteristic results in different coupling with the body from linearly polarized fields. In addition, some studies had suggested that rotating fields might specifically affect melatonin secretion of animals (28) while no change was found for linearly polarized fields (29). Though the evidence was evaluated weak due to the problem of experimental conditions (30), polarization may be taken into account for the health risk assessment of magnetic field exposures.

### Consideration of Library Worker's Exposures

This study has been initiated to contribute to epidemiological studies on IF-EMF exposures of library workers. The results of this study provide useful information about the exposure assessment of library workers due to EAS gates.

Library workers have opportunities to be exposed to IF-EMF from EAS gates when passing through or by the gate. The exposure, in this case, is relatively high-level transient exposure repeated many times a day for a long term. It should be noted that exposures are similar between passing through and passing by the gate. Exposure ratio to basic restriction *ER*_*BR*_ was obtained and it will provide a useful index for this type of exposure, which allows comparing exposures from EAS gates with different operating frequencies and different magnitudes of magnetic fields.

There is another type of exposure for library workers at their working desks. It was found that IF-EMF from the EAS gate spreads a few meters from the gatepost. Library workers can be exposed the whole day long if the working desk is located within a few meters from the gate. This type of exposure can be evaluated based on the magnetic flux density estimated by Equation (3) described in 3.3.

In addition to these exposures, library workers are exposed to magnetic fields from the activator/deactivator of anti-theft magnetic strips for the EAS systems attached to the books (book-check unit, BCU). The device generates strong pulsed magnetic fields. The pulsed fields include IF components. Library workers operate BCU many times a day to cause repeated exposure to the pulsed magnetic field. This type of exposure should also be taken into account when an epidemiological study is planned. This paper does not cover assessment for this type of exposure but we will report it when we get another opportunity.

It is a challenge for exposure assessment of library workers to IF-EMF how to evaluate the combined exposure of those different types of exposures in epidemiological studies.

## Conclusion

We measured IF magnetic fields from five EM-EAS gate models which are commonly used in libraries in Japan. Detailed measurements were performed for two of them in consideration of the phase difference of vector components of magnetic flux density. The polarization of the magnetic field in the gate was investigated with the index of ellipticity. The induced electric field in a human body was numerically calculated for exposures to magnetic fields the two gate models. The results provide a quantitative understanding of the exposures of library workers to the magnetic fields from the EAS gate during passing through or by the gate.

Magnetic field distribution was measured in a large room for one gate model. It was found that the magnetic field was distributed as a function of the horizontal distance to the nearest gatepost. The results provide useful information on how to evaluate the exposure of library workers during their work at the desk.

The 45-point average value *B*_IEC_ defined by IEC standard (19) and CENELEC standard (20) was suggested to be a useful quantity to characterize the magnitude of the magnetic field from the EAS gate. Exposures to different EAS gates are expected to be compared through this quantity without detailed measurements.

The results will provide useful means of exposure assessment of epidemiological studies on the association between IF-EMF exposure and possible health outcomes. The challenges are how to extrapolate the exposure assessed for specific gate models to different models, and how to combine transient exposures due to passing the gate and chronic exposure during work at the desk.

## Data Availability Statement

The original contributions presented in the study are included in the article/supplementary material, further inquiries can be directed to the corresponding author.

## Author Contributions

MI, AA, NK, SY-S, and MT contributed to the study conception and design. KE and KW contributed to measurement. MI and YS contributed to numerical calculation. All authors contributed to the article and approved the submitted version.

## Funding

This work was supported by the Ministry of Internal Affairs and Communications (JPMI10001), Japan.

## Conflict of Interest

The authors declare that the research was conducted in the absence of any commercial or financial relationships that could be construed as a potential conflict of interest.

## Publisher's Note

All claims expressed in this article are solely those of the authors and do not necessarily represent those of their affiliated organizations, or those of the publisher, the editors and the reviewers. Any product that may be evaluated in this article, or claim that may be made by its manufacturer, is not guaranteed or endorsed by the publisher.
